# Soil microbial communities and carbon and nitrogen forms across snow-driven vegetation types in alpine tundra

**DOI:** 10.3389/fmicb.2026.1858414

**Published:** 2026-06-04

**Authors:** Andrea Benech, Emanuele Pintaldi, Samuele Voyron, Laura Gruppuso, Giacomo Marengo, Giampiero Lombardi, Mariangela Girlanda, Michele Freppaz

**Affiliations:** 1 Department of Agricultural, Forest and Food Sciences, University of Turin, Grugliasco, Italy; 2 Department of Life Sciences and Systems Biology, University of Turin, Turin, Italy

**Keywords:** alpine tundra, carbon, functional biodiversity, nitrogen, prokaryotic communities, snow persistence

## Abstract

**Introduction:**

Alpine tundra vegetation, also shaped by snow persistence, is expected to undergo shifts in plant community composition under climate change as snow cover duration declines, with potential consequences for soil biogeochemistry and microbial communities. However, the relationships among snow-driven vegetation types, soil carbon (C) and nitrogen (N) forms, and prokaryotic community functional organization remain poorly understood.

**Methods:**

In this study, we investigated soil C and N forms and the functional organization of prokaryotic communities across three vegetation types shaped by different snow cover duration: snowbed communities dominated by *Salix herbacea* (SB, with long snow cover duration), alpine sedge swards dominated by *Carex curvula* (CC, with short snow cover duration), and intermediate transitional plant assemblages.

**Results:**

C-related forms were mainly associated with vegetation type, with higher C content in CC than in SB. Conversely, mineral N forms varied primarily with timing within the snow free season, with higher concentrations in the early snow free season, indicating different spatial and temporal controls on soil C and N dynamics. Microbial community composition differed among vegetation types, whereas alpha diversity varied little. Despite this, prokaryotic functional groups differed markedly. CC were associated with higher relative abundance of cellulolytic prokaryotes, whereas SB were enriched in N-related functional groups inferred from FAPROTAX assignments, suggesting potential differences in N cycling-related microbial functions among vegetation types.

**Discussion:**

These results show that soil biogeochemistry and microbial functional organization in alpine tundra soils are shaped by both snow-driven vegetation types and timing across the snow free period. Under climate change, reduced snow cover duration and SB contraction may cause the loss of a microhabitat with specific functional biodiversity, with potential consequences for soil C and N cycling.

## 1 Introduction

Alpine tundra represents one of the most fragile and distinctive environments on earth, distributed above the treeline across a wide range of latitudes and geomorphological settings (Körner, 2003). It is characterized by severe climatic conditions such as prolonged snow cover, extended periods with air temperatures below freezing, and short growing seasons, which exert strong selective pressure on both vegetation and belowground communities (Freppaz et al., 2019; Rixen et al., 2022). Within these systems, plants have evolved various physiological and morphological adaptations to cope with extreme conditions, such as dwarf growth forms, clonal propagation, and tolerance to frost and snow burial (Fisk et al., 1998; Carbognani et al., 2014; Körner, 2021).

Among the main factors governing ecosystem functioning in alpine tundra, snow plays a central role (Rixen et al., 2022). Acting as a natural insulator, it buffers soil temperature fluctuations during winter and ensures relatively stable pedoclimatic conditions beneath the snowpack (Brooks and Williams, 1999; Edwards et al., 2007; Freppaz et al., 2018). The timing, depth, and persistence of snow cover regulate not only the soil thermal dynamics but also nutrient availability, organic matter decomposition, and plant phenology (Walker et al., 1993; Groffman et al., 2001; Gavazov et al., 2017; Magnani et al., 2017; Venn and Thomas, 2021).

Within this context, snowbed communities (SB) represent a vegetation type largely controlled by snow persistence. These communities typically develop in topographic depressions or valley bottoms, where snow accumulates in large amounts, assuring stable thermal conditions during winter, and melts only late in the growing season, creating a short window for plant growth and microbial activity (Björk and Molau, 2007; Pintaldi et al., 2022). The delayed snowmelt determines the establishment of highly specialized plant species such as *Salix herbacea* L., which can tolerate both prolonged snow burial and rapid growth once snow disappears (Björk and Molau, 2007; Quaglia et al., 2020; Pintaldi et al., 2022). In contrast, alpine grasslands in wind-exposed topographic positions experience lower snow accumulation and earlier snowmelt, fostering plant communities adapted to longer growing seasons and greater environmental variability, often dominated by species such as *Carex curvula* All. (Matteodo et al., 2016; Bonfanti et al., 2025). This vegetation type mosaic creates strong contrasts in above and belowground dynamics, resulting in pronounced spatial heterogeneity in soil carbon (C) and nitrogen (N) availability and biogeochemical cycling (Benech et al., 2025), with important implications for microbial community composition during the snow free season.

Soils in alpine tundra are generally shallow, stony, and nutrient-poor, with low pH and high organic matter content resulting from slow litter decomposition under cold and often water-saturated conditions (Körner, 2021). In these soils microbial communities play a central role in sustaining ecosystem functioning by driving decomposition, mineralization, and nutrient transformations (Zhou et al., 2023). Vegetation type strongly controls microbial community activity and composition, mediating the effects of seasonal snow cover, soil temperature, and moisture, as well as providing diverse carbon sources, such as plant litter and root exudates, and microhabitats (Shi et al., 2015; Männistö et al., 2024). Under the snowpack, microbial communities remain metabolically active, contributing to organic matter turnover even at sub-zero temperatures (Edwards et al., 2007; Yin et al., 2022). When snow melts, a flush of C and N is typically released into the soil, providing important resources for both microbes and plants (Brooks and Williams, 1999; Walker et al., 1993; Tan et al., 2014). The synchrony between this nutrient release and plant nutrient demand is a central mechanism for ecosystem productivity in alpine tundra, but it is also highly vulnerable to changes in snowmelt timing (Tan et al., 2014; Broadbent et al., 2021). For example, reductions in snow depth or earlier snowmelt have been linked to increased decomposition rates, accelerated N mineralization, and altered bacterial *β*-diversity, with consequences for ecosystem stability and resilience (Zinger et al., 2009; Lazzaro et al., 2015; Ade et al., 2018; Broadbent et al., 2021). Conversely, prolonged snow cover may delay microbial activation and nutrient availability, extending periods of N limitation (Fisk et al., 1998; Venn and Thomas, 2021). Microorganisms catalyze most of the biological transformations of the major elements of life (Falkowski et al., 2008; Madsen, 2011; Wu et al., 2024), and since soil microbes provide nutrients to sustain plant growth, which is strongly nutrient limited in mountain plants, changes in nutrient availability and soil microbial community composition could represent a strong modifier of vegetation shifts in a warming climate (Hagedorn et al., 2019).

Warming temperatures in mountain regions have already led to reductions in snow depth and duration, earlier snowmelt, and higher summer soil temperatures (Beniston et al., 2018; Dorau et al., 2022; Benech et al., 2025). As snow regimes become increasingly unstable, SB are among the most threatened alpine habitats, since their persistence depends directly on prolonged snow cover (Rixen and Wipf, 2017). Their specialist plant species and the microbial processes adapted to late-season soil dynamics are at risk of decline or local extinction if snowpack reduction continues, potentially leading to the contraction or disappearance of these ecosystems (Björk and Molau, 2007; Matteodo et al., 2016). Understanding how SB soils function in terms of C and N dynamics, microbial community composition, and biodiversity is therefore essential for predicting the fate of these ecosystems under global warming. In particular, substantial knowledge gaps remain in understanding how soil C and mineral N forms are linked to microbial community composition across contrasting vegetation types, differently shaped by the snow cover duration. While several studies have examined microbial diversity along elevational gradients (Donhauser and Frey, 2018; Li et al., 2024) or seasonal snow cover patterns (Zinger et al., 2009; Lazzaro et al., 2015; Männistö et al., 2024), few have explicitly connected biogeochemical processes with microbial structure at high elevation (e.g., Broadbent et al., 2021). Moreover, the functional implications of these linkages, such as identifying microbial groups associated with C and N cycling, remain poorly explored. Addressing these gaps is therefore particularly urgent in light of the expected contraction of SB due to reduced snow cover duration, as microbial processes, C and N dynamics, and biodiversity may serve as early indicators of ecosystem responses to a changing climate. The overarching hypothesis is that SB, shaped by late snowmelt, would differ from CC in both biogeochemical and microbial features. Specifically, we expected SB to show different concentrations of C-related forms, distinct seasonal patterns of mineral N availability, and a prokaryotic community composition and inferred functional profile different from those of CC, particularly for microbial groups associated with C degradation and N transformations. To test this hypothesis, we examined how contrasting vegetation communities and snow cover regimes affected C and N forms in topsoils, and whether these differences were associated with shifts in the composition and functionality of soil prokaryotic communities.

## 2 Materials and methods

### 2.1 Study site

**Figure 1:**
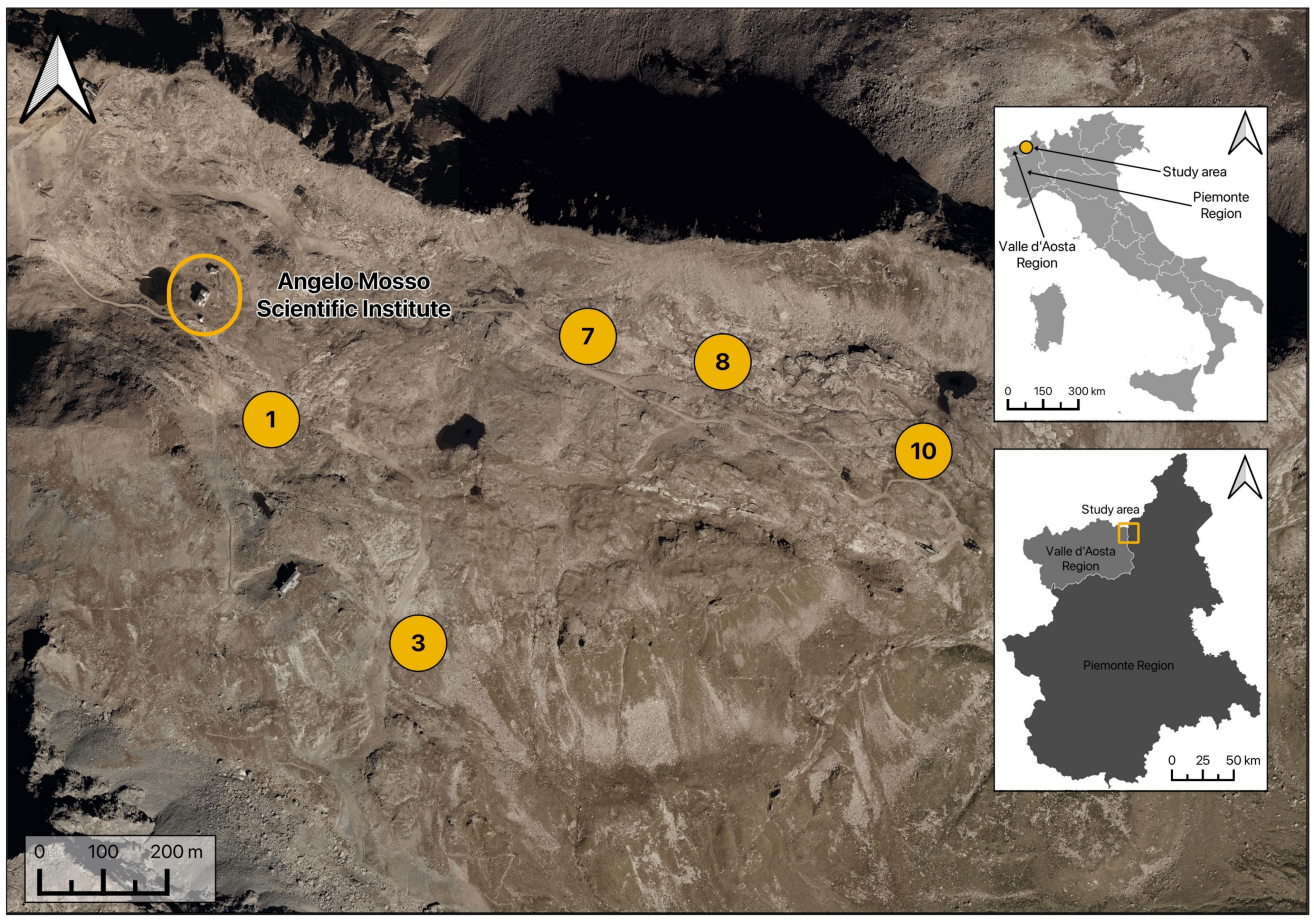
Location of the LTER research study area in Italy (upper inset map) and in the northwestern Italian Alps (lower inset map); aerial overview of the study area (orthoimage year 2018; coordinate system WGS84/UTM 32 N). Numbers, reported according to the LTER site numbering, represent the 5 sites.

**Figure 2:**
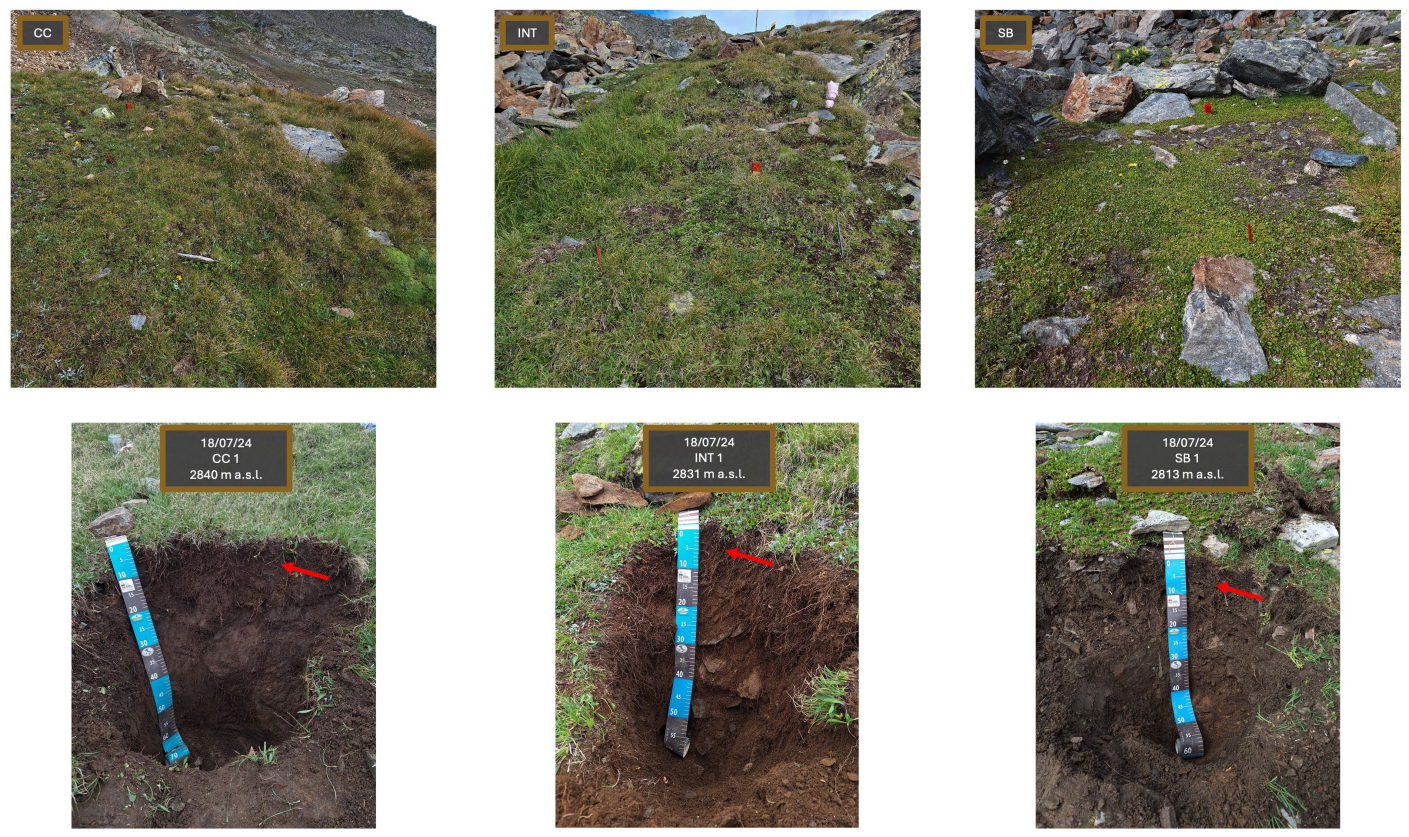
Representative vegetation cover (top) and corresponding soil profiles (bottom) for the three vegetation types: alpine sedge sward dominated by *Carex curvula* (CC, left), intermediate community (INT, middle), and snowbed community (SB, right), at the study site. Red arrows indicate the topsoil layer (0–10 cm).

The study was carried out at the Long Term Ecological Research (LTER) site Istituto Mosso (2650–3,200 m a.s.l.),^1^ located in NW Italy, close to the Monte Rosa Massif (Figure 1). Since 2007, meteorological parameters in the study area have been continuously recorded by an Automatic Weather Station located at 2901 m a.s.l. and operated by the Italian Army (Comando Truppe Alpine - Servizio Meteomont). Between 2007 and 2023, the site experienced a mean annual air temperature of −1.7 °C, an average cumulative annual snowfall of 774 cm, and a mean annual liquid precipitation of approximately 320 mm. Seasonal snow dynamics followed a consistent pattern, with snowpack accumulation typically beginning between late October and early November and snowmelt occurring between late May and early June. The mean snow cover duration was 260 days, ranging from a minimum of 230 to a maximum of 312 days. For the reference period, the melt-out day, defined as the day on which the snow cover had completely disappeared, corresponded to day 188 of the year. Mean topsoil temperature (at 10 cm depth) was 0.1 °C during the snow cover season and increased to 7.8 °C during the snow free season. During the meteorological summer (June–August), the mean air temperature was 5.3 °C. The research was carried out in five high elevation sites, identified according to the LTER site numbering system as sites 1, 3, 7, 8, and 10, and located in the upper part of a glacial plateau at elevations of 2,672, 2,760, 2,773, 2,803, and 2,871 m a.s.l., respectively. The sites are characterized by alpine grasslands belonging to Natura 2000 Habitat 6,150 (“Siliceous alpine and boreal grasslands”). Each site was constituted by three 9 m^2^ plots (15 plots in total): (i) a snowbed community dominated by *Salix herbacea* L. (*Salix herbacea* mean abundance *=* 77.4%) (SB, association *Salicetum herbaceae* Rübel 1911 em. 1933, class *Salicetea herbaceae* Br.-Bl. 1948), (ii) an alpine sedge sward dominated by *Carex curvula* All. (*Carex curvula* mean abundance 78.4 = %) (CC, association *Caricetum curvulae* Rübel 1911, class *Caricetea curvulae* Br.-Bl. 1948) and (iii) an intermediate (INT) plot with transitional plant assemblages (Figure 2). More information about vegetation composition of each vegetation type is available in Supplementary Table 1.

Long term monitoring data from representative historical plots at the LTER site Istituto Mosso indicate contrasting snow cover duration between the two extreme vegetation types, with mean snow cover duration of 261 days in SB and 224 days in CC (2008–2023 period). These values support the characterization of SB and CC as contrasting snow regimes, respectively, although plot-specific snow cover duration was not measured in each sampled plot during the present field campaign.

Topsoil (0–10 cm depth) in each plot was sampled in 2023.

Notably, 2023 was an anomalous year in the north-western European Alps in terms of snow cover duration and mean soil temperature during the snow free, as reported in Benech et al. (2025). In the study area, cumulative snowfall (655 cm) and snow cover duration (243 days) were below the long term average, while air temperature during the snow free season exceeded the average. Thus, the 2023 sampling campaign provides a snapshot of alpine grassland functioning under conditions that may become increasingly common with climate change, notably shorter snow cover periods and warmer snow free seasons (Klein et al., 2016; Beniston et al., 2018; Dorau et al., 2022).

### 2.2 Soil classification and physical and chemical properties

The bedrock was primarily micaschists, with some inclusions of amphibolites and calcschists. Soils were described and classified according to IUSS Working Group WRB (2022). Despite differences in vegetation cover and elevation, the study area showed slight soil variability, resulting in a relatively homogeneous pedological context (Supplementary Table 2). No clear trends in soil type or development were observed among the sites, although some local differences in soil development and carbon content were detected under different vegetation covers. Globally, the sites were characterized by the presence of Umbrisols, Cambisols, and Regosols (Supplementary Table 2), showing only small variations in soil development and physico-chemical properties among sites. However, considering the distribution of soil types related to the vegetation cover (Supplementary Table 2), CC were characterized mainly by Umbrisols (3) and Cambisols (2), whereas INT showed a sharp dominance of Umbrisols (5); otherwise, SB were characterized by the presence of Regosols (2), Cambisols (2) and Umbrisol (1) (Supplementary Table 2).

All soils showed dark-coloured organo-mineral Ah or A horizons (or transitional AB, BA or AC), with weak granular fine structure and abundant roots. These topsoils, sampled for C and N forms, were rich in organic carbon and ranged from 3/5 cm to 40/45 cm in thickness. Subsurface mineral horizons were weakly to moderately developed, commonly transitional (BA, BC), with dark olive, yellowish or brown colours related to the parent material and mainly weak to moderate blocky-subangular structure, stronger in sporadic cambic Bw horizons. Deep mineral horizons were dominated by transitional CA or CB horizons, with single grain or very weak blocky-subangular structure. Transitional CR horizons with weathered bedrock were common, and R layers were frequently detected within the first 40 cm.

Soils under CC were dominated by loamy sand textures, with pH ranging from 4.2 to 5.4 (Table 1). Total organic carbon (TOC) ranged from 13.5 to approximately 165 g kg^−1^ and Total Nitrogen (TN) from 1.3 to 11.8 g kg^−1^; C/N ratios ranged between 10 and 15. INT soils showed loamy sand or sandy loam textures, pH from 4.0 to 5.1, TOC from 4.9 to approximately 140 g kg^−1^, TN from 0.9 to 10.3 g kg^−1^, and C/N ratios between 10 and 18 (Supplementary Table 2). SB soils exhibited loamy sand to sandy textures, pH between 4.2 and 6.1, TOC from approximately 7–8 g kg^−1^ in Bw/AC/CA horizons to over 180 g kg^−1^ in Ah/OH horizons, TN from 0.6 to 12.3 g kg^−1^, and C/N ratios between 8 and 15.

**Table 1:** Mean values and standard deviations of soil pH, total organic carbon (TOC), total nitrogen (TN) and C/N ratio in the three vegetation types (CC, INT, SB).

Vegetation type	pH	TOC (g kg^−1^)	TN (g kg^−1^)	C/N
CC	4.7 ± 0.53	46.7 ± 37.74	3.6 ± 2.67	13 ± 1
INT	4.6 ± 0.35	34.1 ± 28.22	2.6 ± 2.05	13 ± 2
SB	5.3 ± 0.54	22.8 ± 41.20	1.8 ± 2.72	11 ± 2

### 2.3 Soil extractable C and N forms

For soil extractable C and N forms, the topsoil (10 cm depth) in each plot was sampled in triplicate, in 2 periods: July (which we named early snow free season) and September (which we named late snow free season), for a total of 90 samples. In this study, we did not sample topsoil during the winter season. Within 24 h of collection, each soil sample was sieved at 2 mm. An aliquot (10 g) of sieved soil was oven-dried at 105 °C for 24 h to determine the Gravimetric Water Content (GWC). At the same time, another aliquot (20 g) of fresh soil was extracted with 100 mL of KCl 1 M solution. The concentration of dissolved organic carbon (DOC) and total dissolved nitrogen (TDN) in the soil extracts was determined using a TOC analyzer (Elementar, Vario TOC, Hanau, Germany) after filtration through 0.45 μm nylon membrane filters. DOC was used as the main extractable C metric because it represents the soluble and relatively labile fraction of soil organic C that is most directly available for microbial processing over short time scales. Therefore, it was considered appropriate for evaluating vegetation and season related differences in potentially bioavailable C during the snow free period. Extractable ammonium (N-NH₄^+^) concentrations in soil extracts were determined spectrophotometrically (UV-1900i Plus, Shimadzu, Kyoto, Japan) using a modified Berthelot method, based on the reaction with salicylate in the presence of alkaline sodium dichloroisocyanurate (Crooke and Simpson., 1971). Extractable nitrate (N-NO₃^−^) concentrations were measured spectrophotometrically (UV-1900i Plus, Shimadzu, Kyoto, Japan) following the Griess reaction as described by Mulvaney (1996) and modified by Cucu et al. (2014). DON was calculated as TDN - (N-NH_4_^+^ + N-NO_3_^−^).

### 2.4 DNA extraction and sequencing

For DNA extraction and sequencing, the topsoil was sampled in quadruplicate at each plot and in each sampling period, resulting in a total of 120 samples. Prior to DNA extraction, collected soil samples were sieved to remove roots and small rocks. Environmental DNA (eDNA) was then extracted from 100 mg of sieved soil using the DNeasy® PowerSoil® Pro Kit (QIAGEN), following the manufacturer’s instructions.

To investigate prokaryotic communities, a DNA metabarcoding approach was employed by amplifying the V4 region of the 16S ribosomal RNA (rRNA) gene using the primer pair 515fB (5′–GTGYCAGCMGCCGCGGTAA–3′) and 806rB (5′–GGACTACNVGGGTWTCTAAT–3′) (Parada et al., 2016; Apprill et al., 2015).

Amplification of the 16S rRNA gene was performed using the Invitrogen Platinum II HotStart PCR Master Mix (Thermo Fisher Scientific, Waltham, MA, USA). The thermal cycling protocol consisted of an initial denaturation at 94 °C for 3 min, followed by 35 cycles of denaturation at 94 °C for 45 s, annealing at 55 °C for 60 s, and extension at 72 °C for 90 s. A final extension step was performed at 72 °C for 10 min.

Each sample was amplified in triplicate, and the resulting PCR products were pooled before purification. The PCR triplicates represented technical amplification replicates and were pooled prior to sequencing; only biological samples were considered in downstream analyses. Pooled amplicons were purified using the Wizard® SV Gel and PCR Clean-Up System (Promega), according to the manufacturer’s protocol. DNA concentration was quantified using the Qubit™ dsDNA BR Assay Kit with the Qubit™ 2.0 Fluorometer (Thermo Fisher Scientific). Purified libraries were then sent to IGA Technology Services (Udine, Italy) for paired-end sequencing (2 × 250 bp) on an Illumina NovaSeq platform.

### 2.5 Bioinformatics and statistical analysis

Differences in soil C and N concentrations among vegetation types (CC, INT, SB) and between early and late snow free seasons were analysed using generalized linear mixed-effects models. For each response variable, vegetation community, season, and their interaction were included as fixed effects, while transect identity was included as a random effect to account for repeated measurements. Since all response variables were positive and continuous, both Gaussian models and Gamma models with a log link were fitted and compared using Akaike’s Information Criterion (AIC). Final inference was based on Gamma mixed-effects models with a log link, as these models showed the lowest AIC for all response variables. Model significance was assessed using joint tests of the fixed effects. Model assumptions were evaluated using simulation-based residual diagnostics, including tests for residual uniformity, dispersion, and outliers. Post-hoc pairwise comparisons were performed on estimated marginal means using Tukey’s adjustment.

Sequencing adapters and primers were removed prior to downstream analysis using the microbiome bioinformatics platform QIIME2 (Quantitative Insights Into Microbial Ecology 2) (Bolyen et al., 2019). Denoising and quality filtering, including chimera removal, were performed using the DADA2 plugin (Callahan et al., 2016) via the q*iime dada2 denoise-paired command*, with chimera detection set to the *consensus* method. The resulting denoised reads were retained as Amplicon Sequence Variants (ASVs), which were used for all downstream analyses.

Taxonomic classification of the prokaryotic community was performed using the SILVA 138 reference database, based on full-length 16S rRNA gene sequences (Bokulich et al., 2018; Robeson et al., 2021). Phylogenetic trees were constructed with the QIIME2 plugin *qiime phylogeny align-to-tree-mafft-fasttree.* The main outputs from the QIIME2 pipeline: t*axonomy.qza, feature_table.qza*, and *rooted-tree.qza,* together with the representative sequences FASTA and the corresponding metadata files, were imported into RStudio (R Core Team, 2023). These data were used to construct *phyloseq* objects using the *qiime2R* package (v0.99.6) (Bisanz, 2018), providing a framework for subsequent statistical analyses and visualization of the microbial community structure.

To allow comparisons among samples with not-uniform sequencing depth, ASV tables were normalized using the rarefy_even_depth function of the R package phyloseq v.1.36.0 (McMurdie and Holmes, 2013), thereby standardizing sequencing effort and enabling direct comparisons of microbial communities among samples. The *rarecurve function* of the R package *vegan* v.2.6–2 (Oksanen et al., 2020) was used to obtain rarefaction curves of the rarefied ASV table. Taxonomical plots were obtained using the R packages *ggplot2* v.3.5.1 (Wickham, 2016) and ggh4x v.0.2.8 (Van den Brand, 2024).

Shannon alpha diversity index was calculated using the estimate_richness function of the R package phyloseq. The effects of vegetation type and timing during the snow free season on alpha diversity were evaluated using analysis of variance (ANOVA).

Microbial community variations across different conditions were assessed using distance-based redundancy analysis (dbRDA) with the Bray–Curtis dissimilarity index, as implemented in the *microeco* R package (v1.5.0) (Liu et al., 2021). The dbRDA ordination included continuous biogeochemical and environmental variables, including DOC, DON, N-NH₄^+^, N-NO₃^−^ and GWC, in order to evaluate the contribution of environmental gradients to microbial community differentiation. Functional annotation of prokaryotic taxa was performed using the FAPROTAX database (v1.2.6) (Louca et al., 2016), likewise integrated into the *microeco* package. Differences in prokaryotic community composition at the class level among vegetation types were assessed using PERMANOVA based on Bray-Curtis dissimilarities (9,999 permutations), as implemented in the *vegan* R package. Prior to analysis, the ASV table was agglomerated at the class rank and transformed to relative abundances per sample. To evaluate differences in the relative abundance of individual dominant classes, only taxa with a mean relative abundance > 1% across all samples were considered. For each class, differences among vegetation types were tested using the Kruskal–Wallis test, followed by Dunn’s *post hoc* comparisons with Benjamini–Hochberg correction for multiple testing. Statistical significance was accepted at *p* < 0.05.

The dataset generated in this study is publicly available in the NCBI Sequence Read Archive (SRA;https://submit.ncbi.nlm.nih.gov/subs/sra/SUB16029447, accessed on 27 February 2026) under BioProject accession number PRJNA1429086.

## 3 Results

### 3.1 Soil C and N forms

**Figure 3:**
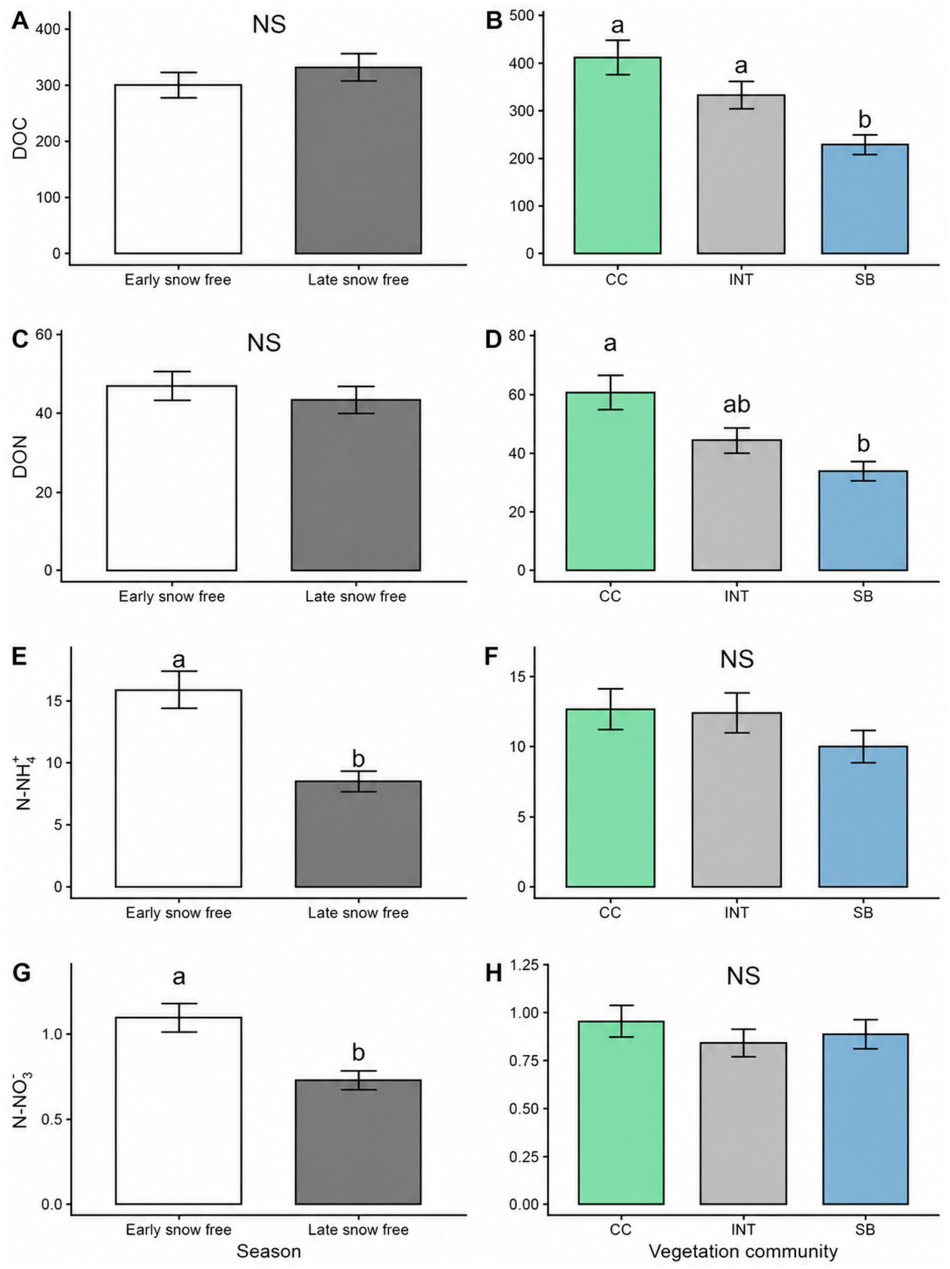
Model-estimated marginal means ± standard errors (*n* = 30) of soil C and N forms by season (white bars for early snow free season and dark grey bars for late snow free season; Subfigures **A, C, E, G**) and vegetation communities (CC, green; INT, light grey; SB, blue; Subfigures **B, D, F, H**). Estimates were obtained from generalized linear mixed-effects models and back-transformed to the response scale. Different letters indicate significant *post-hoc* differences; “NS” indicates not-significant effects.

DOC concentrations differed significantly among vegetation types (*p* = 0.001), while no significant effects of season or interaction between vegetation types and season were detected. *Post hoc* comparisons confirmed significantly higher DOC concentrations in CC compared to SB (*p* = 0.001), whereas differences between CC and INT were not significant (*p* > 0.05) (Figures 3A,B).

DON concentrations were significantly influenced by vegetation types (*p* = 0.035), while neither season nor the interaction between vegetation types and season had a significant effect (*p* > 0.05). Post hoc comparisons indicated that the vegetation type effect was mainly driven by higher DON in CC than in INT and SB (*p* = 0.050). No early-late snow free season differences were detected within any vegetation types (*p* > 0.05) (Figures 3C,D).

Soil mineral N concentrations (N-NH_4_^+^ and N-NO_3_^−^) were significantly influenced by season (*p* = 0.001 for both variables), whereas no significant effects of vegetation types or interaction between vegetation types and season were observed (Figures 3E,F). N-NH_4_^+^ concentrations exceeded those of N-NO_3_^−^. Across all vegetation types, both N-NH_4_^+^ and N-NO_3_^−^ concentrations were significantly higher during the early snow free period than during the late snow free period (*p* = 0.001) (Figures 3G,H). Regarding GWC, INT exhibited the highest values (mean of 27%), followed by CC (mean of 26%) and SB (mean of 22%).

### 3.2 Soil microbial community and C and N functional groups

A total of 116 biological DNA samples were retained after sequencing quality control and bioinformatic processing, yielding 667,812 high-quality sequences. To allow direct comparison among samples and minimize biases related to uneven sequencing depth, the dataset was rarefied to 5,757 sequences per sample. Following rarefaction, the final dataset consisted of 15,921 ASVs. The effects of rarefaction on the ASV table were evaluated using rarefaction curves, which showed a general tendency toward stabilization across samples (Supplementary Figure 1), indicating adequate sequencing depth.

Across vegetation types, prokaryotic communities were overwhelmingly dominated by Bacteria, accounting for 98.0%, 99.0%, and 98.0% of total sequences in CC, INT, and SB plots, respectively, whereas (consistently with the use of universal primers for the 16S rRNA gene; Bahram et al., 2019) Archaea represented only 2.0%, 1.0%, and 2.0%.

**Figure 4:**
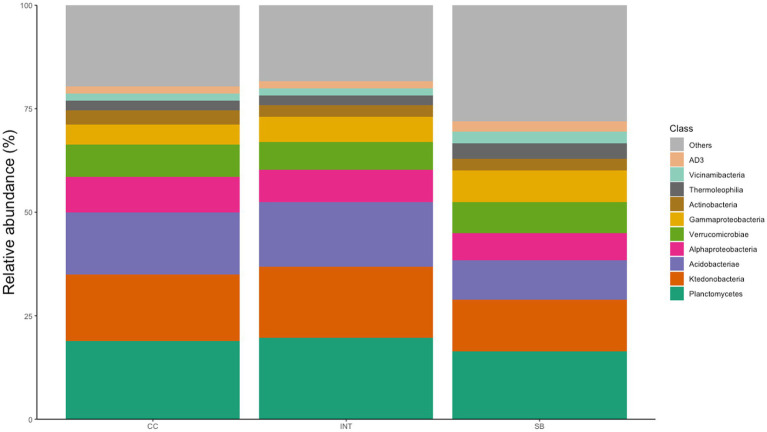
Relative abundance (%) of bacterial classes in the three vegetation types (CC, left; INT, middle; SB, right). Relative abundances are averaged across the two sampling periods (early and late snow free season).

Similar dominant bacterial classes were observed across the three vegetation groups (Figure 4; Supplementary Figure 2), although their relative contributions differed among plots. Across all vegetation types, Planctomycetes (Planctomycetota), Ktedonobacteria (Chloroflexota), and Acidobacteriae (Acidobacteriota) represented the most abundant classes, collectively accounting for a substantial fraction of the community. CC plots were characterized by relatively high abundances of Planctomycetes (Planctomycetota; 18.8%), Ktedonobacteria (Chloroflexota; 16.3%), and Acidobacteriae (Acidobacteriota; 15.1%). INT plots displayed a comparable composition, with Planctomycetes (19.5%), Ktedonobacteria (17.2%), and Acidobacteriae (15.6%) as the dominant groups. In SB plots, Planctomycetes (16.2%) and Ktedonobacteria (12.5%) remained among the most abundant classes, whereas Acidobacteriae decreased to 9.7%. Gammaproteobacteria (7.7%), Verrucomicrobiae (7.4%), Alphaproteobacteria (6.6%), Thermoleophilia (3.7%), and Vicinamibacteria (2.7%) remained relatively abundant or showed proportionally higher abundances in SB compared with the other vegetation groups. Taxa grouped as “Others” accounted for approximately 28–29% of the community in SB plots, compared to 18% and 20% in CC and INT, respectively.

These compositional differences were statistically supported by PERMANOVA analysis at the class level based on Bray-Curtis dissimilarities. Pairwise comparisons indicated that SB differed significantly from both CC (R^2^ = 0.113, *F* = 9.69, *p* = 0.0001) and INT (R^2^ = 0.129, *F* = 11.21, *p* = 0.0001), whereas no significant difference was detected between CC and INT (R^2^ = 0.016, *F* = 1.20, *p* = 0.264). At the individual class level, several dominant taxa showed significant differences among vegetation types (Kruskal–Wallis test followed by Dunn’s *post hoc* test with Benjamini-Hochberg correction; Supplementary Figure 2), particularly Gammaproteobacteria, Thermoleophilia, Vicinamibacteria, Anaerolineae, Bacteroidia, and WPS-2, further supporting the distinct compositional profile observed in SB plots.

**Figure 5:**
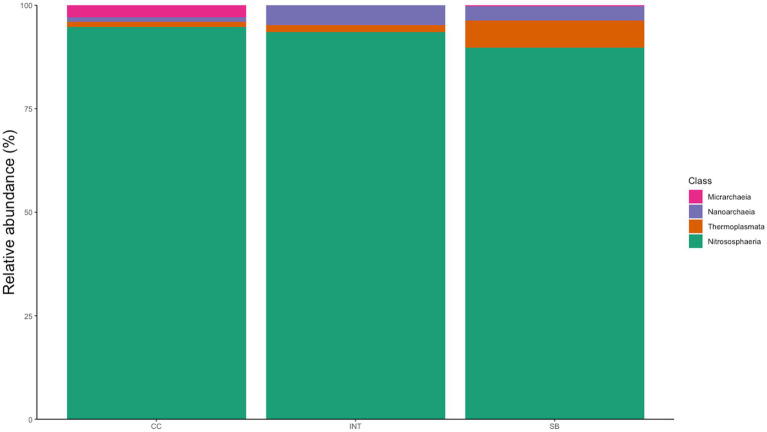
Relative abundance (%) of archaeal classes in the three vegetation types (CC, left; INT, middle; SB, right). Relative abundances are averaged across the two sampling periods (early and late snow free season).

Archaeal communities were largely dominated by Nitrososphaeria across all vegetation types (Figure 5), accounting for approximately 90% of archaeal sequences. Minor contributions from Thermoplasmata, Nanoarchaeia, and Micrarchaeia were also detected. However, no significant differences in archaeal community composition among vegetation types were observed based on PERMANOVA analysis (*p* > 0.05), indicating a relatively stable archaeal assemblage across the studied alpine tundra communities.”

**Figure 6:**
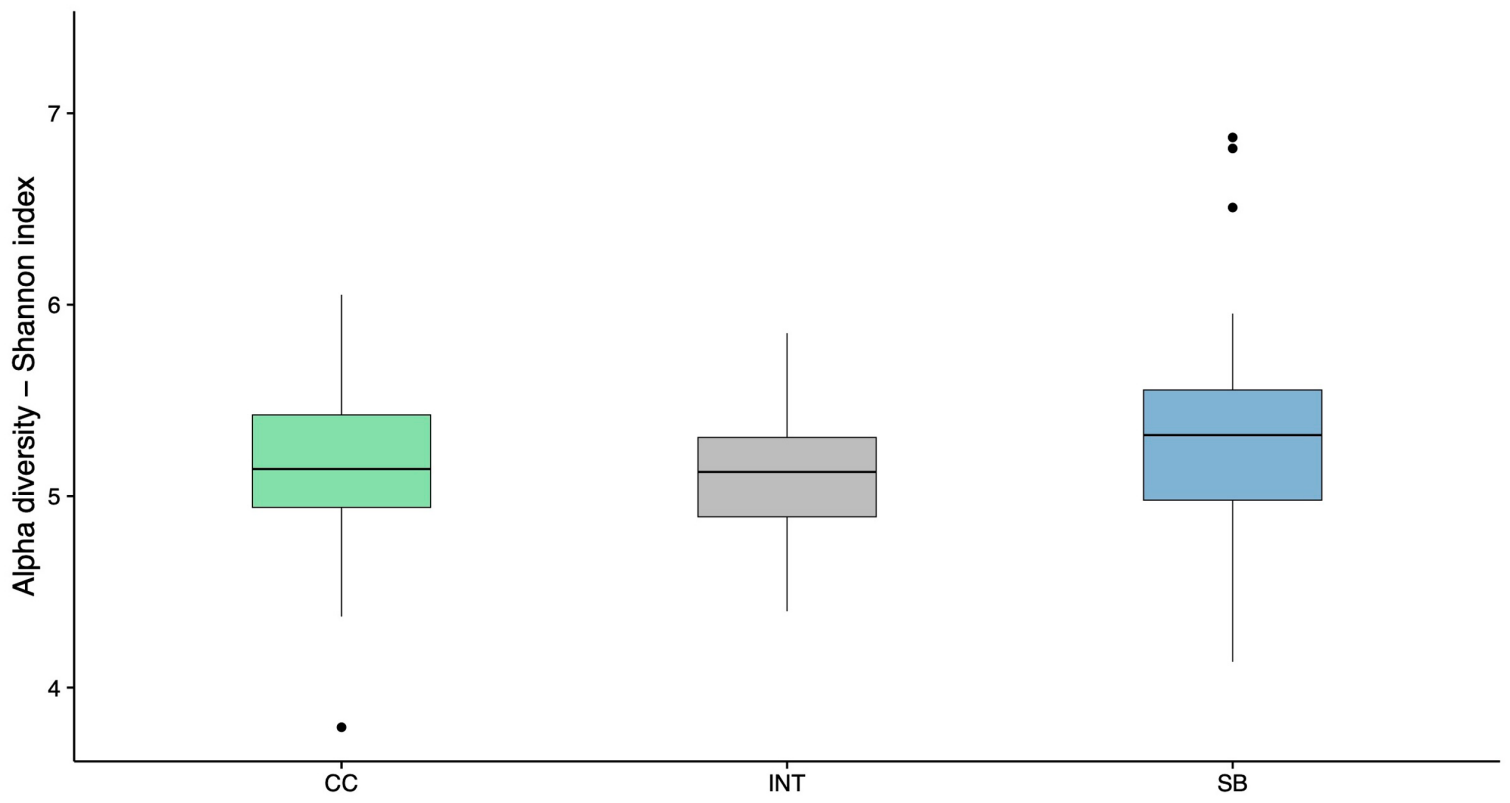
Shannon index in the three vegetation types (CC, green; INT, grey; SB, blue), including samples from both sampling periods (early and late snow free season).

Prokaryotic *α*-diversity, expressed as the Shannon index, did not differ significantly among vegetation types (*p > 0.*05, Figure 6).

**Figure 7:**
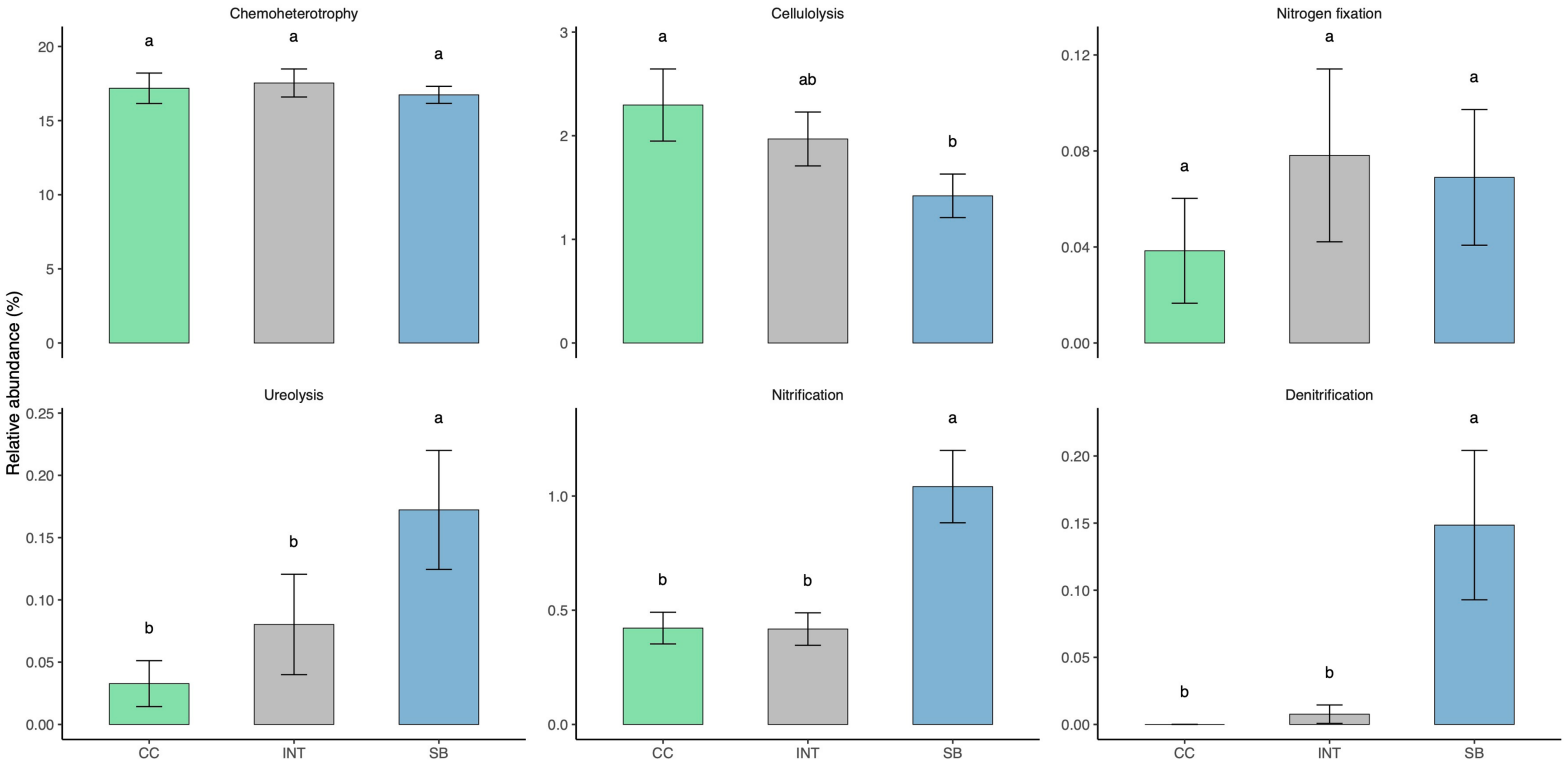
Relative abundance (%) of selected microbial functional groups (chemoheterotrophy, cellulolysis, nitrogen fixation, ureolysis, nitrification, and denitrification) across the three vegetation types (CC, green; INT, grey; SB, blue). Bars represent the mean across the two sampling periods (early and late snow free season). Error bars represent the standard deviation (*n* = 40 for SB, *n* = 38 for INT and CC). Different letters denote significant differences among vegetation types within each functional group (*p* < 0.05).

Differences among prokaryotic communities associated with the three vegetation types emerged across multiple putative C and N related functional groups inferred from FAPROTAX assignments (Figure 7). Taxa assigned to chemoheterotrophic functional groups showed the highest relative abundance overall and did not differ significantly among vegetation types (*p* > 0.05). Occurrence of taxa assigned to cellulolytic functional groups varied among vegetation types, with higher values in CC than in SB (*p* < 0.05), while INT displayed intermediate values. In contrast, taxa assigned to putative N cycling functional groups showed an opposite trend: the relative abundance of taxa potentially associated with nitrification and denitrification, as well as ureolysis, was significantly higher in SB than in CC and INT (*p* < 0.05). Taxa assigned to nitrogen fixation did not differ significantly among vegetation types (*p* > 0.05).

### 3.3 Correlation between soil C and N forms and temporal dynamics of microbial communities

**Figure 8:**
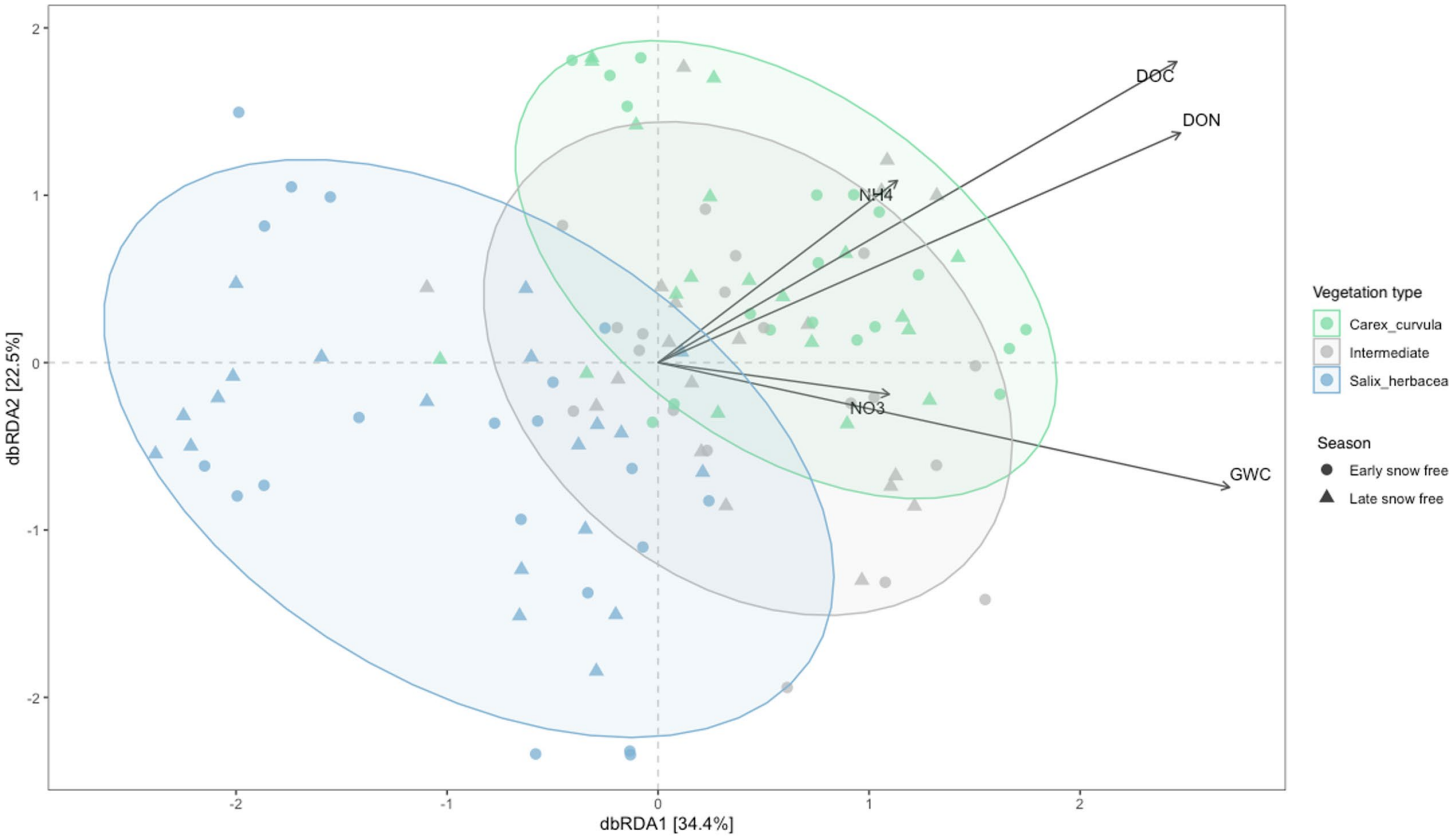
Distance-based redundancy analysis (dbRDA) ordination of microbial community composition across vegetation types and sampling periods. Points represent individual samples and are colored by vegetation type (CC, green; INT, grey; SB, blue), while symbols indicate sampling period (circles, early snow free season; triangles, late snow free season). Arrows represent significant vectors (DOC, DON, N-NH_4_^+^, N-NO_3_^−^, GWC). Percentages on axes indicate the proportion of constrained variation explained by each dbRDA axis.

The distance-based redundancy analysis (dbRDA) (Figure 8) showed a constrained structure of prokaryotic community composition across samples. The first two constrained axes explained 56.9% of the constrained variation, with dbRDA1 accounting for 34.4% and dbRDA2 for 22.5%. Vegetation types separated mainly along dbRDA1: SB samples were distributed mostly on the negative side of the axis, whereas CC and INT samples were shifted toward positive scores, with partial overlap between CC and INT. Among the environmental variables included in the ordination, DOC, DON, GWC, and N-NH₄^+^ were significant vectors (*p* < 0.05), whereas N-NO₃^−^ was not significant (*p* > 0.05). DOC and DON were oriented toward the positive side of dbRDA1, corresponding to the distribution of most CC and INT samples. GWC showed a similar vector length to DOC and DON and was oriented toward positive dbRDA1 and negative dbRDA2 scores. N-NH₄^+^ was also oriented toward the positive side of dbRDA1, although its vector was shorter than those of DOC, DON, and GWC. No clear separation between early and late snow free season samples was observed in the dbRDA. Samples from the two sampling periods were largely intermingled within each vegetation type.

## 4 Discussion

### 4.1 Soil C and N forms

The significant differences in DOC concentrations among vegetation types (Figure 3) align with the results presented in Benech et al. (2025) suggesting that DOC in alpine tundra soils is firstly driven by the vegetation cover rather than short-term temporal variability. The higher DOC concentrations in CC compared to SB are consistent with differences in vegetation composition, organic matter inputs, and soil organic matter accumulation, all of which are known to influence DOC production and availability (Zhao et al., 2022). According to the higher soil C content (Table 1), CC sustain a more continuous supply of plant-derived organic inputs, through persistent litter and substantial belowground inputs (Klug-Pumpel, 1981), thereby supporting greater SOM accumulation and a larger DOC source. Conversely, SB are constrained by late snowmelt and a shorter growing season, which limit plant production and reduces the release of fresh organic material to the soil (Wijk, 1986; Björk and Molau, 2007). However, DOC should not be interpreted as representative of the whole soil C pool. It accounts only for the dissolved and relatively mobile fraction of organic C, whereas particulate organic matter, mineral-associated organic matter, microbial biomass C, and total organic C may respond differently to vegetation type and snow cover duration. In particular, DOC concentrations reflect a balance between production, microbial uptake, sorption, and hydrological transport (Kaiser and Kalbitz, 2012), and therefore provide information on short term C availability rather than on long term soil C storage. For this reason, our interpretation of C dynamics is restricted to the extractable and potentially bioavailable C fraction measured during the snow free season.

DON exhibited spatial patterns similar to DOC, indicating that even DON is primarily shaped by site-specific differences in vegetation composition rather than short-term temporal variability (Figure 3). In cold and alpine ecosystems, DON can represent a substantial fraction of dissolved N and is tightly coupled to the size and composition of the dissolved organic matter pool (Neff et al., 2003; Hood et al., 2003). This pattern is also consistent with the higher total N in CC (Table 1), which suggests a larger soil organic N reservoir that can sustain DON availability. The parallel enrichment of DOC and DON in CC therefore points to a larger pool of soluble organic matter, including nitrogen containing compounds produced during litter and SOM decomposition and microbial processing (Kalbitz et al., 2000).

In contrast to DOC and DON, mineral N availability is primarily controlled by timing during the snow free season. Across vegetation types, both N-NH_4_^+^ and N-NO_3_^−^ concentrations are higher during the early snow free period and decrease towards the late snow free period, consistent with the snowmelt pulse of inorganic N (Figure 3). This pulse is generally attributed to (i) nutrient release from the snowpack (ionic pulse), which represents an important source of dissolved inorganic and organic N (Brooks and Williams, 1999; Filippa et al., 2010; Westergaard-Nielsen et al., 2020), and to (ii) sustained microbial activity under the snowpack and rapid mobilization of mineral N upon thaw, followed by progressive biological demand during the growing season (Lipson et al., 1999; Broadbent et al., 2021).

Together, these results highlight a decoupling between spatial controls on DOC and temporal controls on mineral N, highlighting the need to consider both dimensions when assessing soil biogeochemical functioning in alpine grasslands under changing snow cover conditions.

### 4.2 Soil microbial community and C and N functional groups

Soil microbial communities exhibit regional differences, and only limited overlap of prokaryotic spectra and dominance patterns was found with either SB or CC soils in another Italian region (Sannino et al., 2022; D'Alò et al., 2023). In this study, prokaryotic sequences were analysed using an ASV-based approach following DADA2 denoising, allowing high-resolution characterization of microbial community composition across vegetation types. Despite the taxonomic resolution provided by ASVs, the analyses focused primarily on broad ecological and functional differentiation among vegetation communities rather than on fine-scale microdiversity patterns. However, the prevalence of ammonium-oxidizing microbes (such as archaea and bacteria in Thermoproteota and Acidobacteriota, respectively) is common in high elevation soils, and it likely stems from their adaptation to the nitrogen-poor conditions characteristic of these environments (Praeg et al., 2025).

The Shannon index (Figure 6) showed only limited variation among vegetation types, indicating that prokaryotic alpha diversity remained broadly comparable across the vegetation types. This suggests that the transition from CC to SB does not alter within-community diversity in quantitative terms. However, similar alpha-diversity values do not necessarily imply similar community functioning (Knelman and Nemergut, 2014). Thus, communities can maintain comparable levels of alpha diversity while differing substantially in their community organization, a distinction that becomes particularly relevant when considering functional biodiversity.

The distribution of prokaryotic functional groups differed significantly among vegetation types (Figure 7). This indicates marked differences in functional biodiversity despite the limited variation in alpha diversity. C-related groups followed the strong C gradient across vegetation types. The higher abundance of cellulolytic prokaryotes in CC (with INT perfectly intermediate) was consistent with the larger organic C pools (Table 1) and higher DOC found under CC (Figure 3), because sustained plant inputs and greater SOM accumulation typically increase the availability of polymeric substrates (e.g., cellulose) (Ai et al., 2023; Chen et al., 2025) and promote the production/release of DOM during decomposition and microbial processing (Kaiser and Kalbitz, 2012). Conversely, although chemoheterotrophic prokaryotes were dominant in relative abundance across all functional groups, they did not differ among communities, suggesting that broad heterotrophic metabolism is ubiquitous across these alpine soils, while the capacity for specific C polymers breakdown (i.e., cellulolysis) is the component that best discriminates the SB-CC transition in organic matter availability.

In contrast, SB soils were enriched in prokaryotes associated with N-related functions (nitrification, denitrification, and ureolysis) inferred from FAPROTAX assignments (Figure 7), although showing N-NH_4_^+^ and N-NO_3_^−^ concentrations comparable to the other vegetation communities (Figure 3).

This apparent mismatch suggests that the relative abundance of taxa potentially associated with N transformations does not necessarily correspond to larger standing pools of inorganic N. One possible explanation is that inorganic N produced or transformed in SB soils does not accumulate because it is consumed or removed just as quickly, preventing its accumulation in the measured soil extracts. However, because we did not directly measure N turnover rates, microbial immobilization, plant uptake, leaching, or gaseous N losses, this interpretation remains hypothetical. In this sense, our results may be viewed as broadly compatible with the “missing N” concept proposed for cold and nutrient-limited ecosystems (e.g., Yanai et al., 2013; Buckeridge et al., 2023), but they do not provide direct evidence for this mechanism. Among the N-related functional groups inferred by FAPROTAX, taxa assigned to nitrification were the most represented in SB (Figure 7), suggesting the potential occurrence of enhanced N-NH_4_^+^ oxidation capacity. This pattern is consistent with the hypothesis that, in SB, N-NH_4_^+^ produced via mineralization and/or released during snowmelt can be rapidly converted to N-NO_3_^−^. In alpine tundra soils, this mineral N can then be rapidly partitioned among microbial immobilization, plant uptake and leaching losses (Schmidt et al., 2007; Muratore et al., 2021; Vincent et al., 2025), helping to explain why inorganic N pools remain low despite high transformation potential. Microbial immobilization in cold ecosystems tends to be stronger late in the growing season and during winter, when mineral N can be incorporated into microbial biomass and later released (Bardgett et al., 2002; Schmidt et al., 2007).

However, because our sampling did not target the snow cover season when microbial immobilization is expected to peak, its contribution at the time of measurements was likely limited. Consequently, in our study it is reasonable that plant uptake plays a dominant role in keeping inorganic N pools low and similar across communities, particularly in SB where the growing season is shorter and plants must acquire nutrients within a narrower time window (Jaeger et al., 1999). By contrast, a longer growing season in CC may spread N demand over time, making early-season depletion less pronounced. Beyond biological demand, hydrologic transport provides an additional pathway by which mineral N, especially N-NO_3_^−^, can be transported to deeper soil horizons and leached from the soil (Owen and Jones, 2001; Latifah et al., 2017; Vincent et al., 2025). Leaching may be more important in SB because these sites are typically wetter than CC, especially in the early snow free season after the snow melting (Björk and Molau, 2007; Sedlacek et al., 2015). Specifically, even at similar concentrations, total N losses can be higher when leaching volumes are larger; for this reason, soil solution measurements that combine concentrations with flux estimates would help quantify hydrologic N export via leaching. Moreover, the same wet conditions that enhance leaching can also promote oxygen limitation, creating opportunities for N gases losses through denitrification. Indeed, an enrichment in SB denitrification was observed (Figure 7), suggesting that a fraction of inorganic N may also be lost as gases, especially during anaerobic phases. In alpine tundra environments, such conditions are most likely during and immediately after snowmelt, when soils can become saturated and support localized zones of elevated denitrification activity (Morse et al., 2015).

### 4.3 Correlation between soil C and N forms and microbial community composition

The dbRDA ordination (Figure 8) indicates that a substantial fraction of the variation in prokaryotic community composition correlates with the measured soil C and N forms and gravimetric water content. Vegetation types separated primarily along the first axis, with SB communities clearly distinct from those associated with CC and INT. The partial overlap between CC and INT supports the existence of an ecological continuum, suggesting that the transition between the two grassland communities corresponds to the establishment of microbial communities that resemble CC more than SB under intermediate conditions.

The orientation of environmental vectors suggests that DOM represents the dominant gradient associated with microbial community differentiation, because both DOC and DON were significantly aligned with the positive direction of dbRDA1, where most CC and INT samples were distributed. This matches the biogeochemical evidence that DOC and DON vary mainly among vegetation types, with higher values in CC and INT and lower values in SB. Together, these results suggest that differences in vegetation driven organic inputs and SOM availability are major factors structuring prokaryotic communities across the vegetation gradient. Since DOC and DON are closely linked to soil organic matter inputs, litter decomposition, root-derived substrates, and microbial processing, their association with microbial community composition likely reflects vegetation-associated differences in the availability of soluble organic substrates, as reported by several studies (e.g., Liu et al., 2020; Zhao et al., 2021; Smith et al., 2024).

GWC also contributed to the distribution of microbial communities, with a significant vector oriented toward the positive side of dbRDA1 and the negative side of dbRDA2. This pattern indicates that soil moisture was an additional edaphic factor associated with microbial community composition across the vegetation-soil gradient. Indeed, soil bacterial communities are particularly impacted by variation in soil moisture, due to direct and indirect effects via changes in vegetation composition (De Vries et al., 2018; Bogati and Walczak, 2022). The orientation of GWC vector, partly consistent with the distribution of CC and INT samples, suggests that differences in soil water availability may have contributed to the observed community structure together with dissolved organic matter. This is ecologically plausible in alpine tundra soils, where soil moisture can influence oxygen availability, substrate diffusion, microbial metabolism, and the balance between aerobic and anaerobic processes (Drenovsky et al., 2004; Frindte et al., 2019). However, the GWC vector did not correspond to a distinct separation of SB from the other vegetation types on its own, indicating that soil moisture should be interpreted as part of a broader set of covarying edaphic conditions rather than as an isolated driver.

In contrast, inorganic N forms were less strongly associated with microbial community differentiation. Although N-NH₄^+^ was a significant vector, its shorter length suggests a weaker contribution to the ordination structure compared with DOC, DON, and GWC. N-NO₃^−^ was not significant. These considerations are consistent with the observation that mineral N pools were primarily controlled by season across vegetation types, generally decreasing from the early to the late snow free period. However, the dbRDA did not show a clear separation between early and late snow free season samples, indicating that seasonal changes in inorganic N availability did not translate into a strong seasonal shift in prokaryotic community composition. Thus, during the snow free season, microbial community differentiation among vegetation types appears to be more closely linked to spatial variability in soluble organic matter and soil moisture rather than to instantaneous mineral N pool sizes.

Recent studies support the relevance of these patterns under ongoing climate change. Experimental snow drought in subalpine grasslands has been shown to alter soil microbial communities and greenhouse gas fluxes, highlighting the sensitivity of microbial processes to reduced winter snow protection (Bonfanti et al., 2026). In addition, Broadbent et al. (2024) showed that reduced snow cover and vegetation shifts can interactively disrupt the seasonal coupling between plant and soil microbial N cycling in alpine grasslands. In this context, the differences observed here among CC, INT, and SB may help anticipate how future reductions in snow cover duration and associated vegetation changes could be accompanied by shifts in soil C and N availability and microbial community composition.

Overall, the ordination suggests that prokaryotic community structure in these alpine tundra soils was mainly associated with vegetation type and GWC. Vegetation type was associated with differences in DOC and DON, reflecting variation in soluble organic matter availability among CC, INT, and SB. This vegetation-associated gradient clearly separated SB microbial communities from those of CC and INT, with INT showing greater similarity to CC. GWC showed an association of comparable importance with microbial community distribution, indicating that soil moisture was also a key edaphic factor structuring prokaryotic communities.

These patterns should be interpreted with caution because vegetation type was not independent from other environmental factors. Differences among CC, INT, and SB may covary with soil development, hydrological conditions, organic matter inputs, rooting patterns, and snow persistence. Soil type was evaluated but not retained in the final dbRDA because it was unevenly distributed among vegetation types, while TOC and TN were excluded because of strong collinearity. In addition, plot-level measurements of soil depth, topographic position, snow-cover duration or snowmelt timing, soil temperature, root biomass, litter quality, and detailed hydrological parameters were not available. Thus, while the observed patterns robustly describe differences in soil biogeochemistry and microbial community composition among vegetation types, their interpretation should consider the covariation between vegetation and broader soil environmental conditions.

In conclusion, our findings highlight that soil biogeochemistry in alpine tundra was associated with distinct spatial and temporal factors. Vegetation type primarily structures C-related forms (particularly DOC and DON), whereas mineral N forms varied primarily with timing within the snow free season. Alpine tundra vegetation types hosted prokaryotic communities with marked differences in inferred functional biodiversity, particularly in putative C and N cycling functions, even when alpha diversity varied little. Notably, SB soils were associated with higher relative abundance of taxa assigned to N-related functional groups, suggesting that they may represent functionally distinct microbial microhabitats compared with the other vegetation communities. Together, these results show that vegetation types, largely controlled by snow persistence, may shape both soil biogeochemistry and microbial functional organization. Given the expected climate-driven shifts in vegetation associated with reduced snow cover duration, the contraction of SB habitats, hotspots of high biodiversity in alpine ecosystems, may alter the functionality of distinct soil microbial niches, with potentially significant consequences for alpine soil C and N cycling.

## Data Availability

The datasets presented in this study can be found in online repositories. The names of the repository/repositories and accession number(s) can be found below: https://www.ncbi.nlm.nih.gov/, PRJNA1429086.

## Footnotes

1
https://deims.org/17210eba-d832-4759-89fa-9ff127cbdf6e
