# Supplementary material for: Soil microbial communities and carbon and nitrogen forms across snow-driven vegetation types in alpine tundra
Source: Front Microbiol. 2026 Jun 4;17:1858414. doi: 10.3389/fmicb.2026.1858414 (PMC13275723; doi:10.3389/fmicb.2026.1858414)
Supplement: Supplementary file 1 [file Table_1.DOCX]

**Soil microbial communities and carbon and nitrogen forms across snow-driven vegetation types in alpine tundra**

Andrea Benech^1^, Emanuele Pintaldi^1*^, Samuele Voyron^2^, Laura Gruppuso^2^, Giacomo Marengo^1^, Giampiero Lombardi^1^, Mariangela Girlanda^2^, Michele Freppaz^1^

^1^Department of Agricultural, Forest and Food Sciences, University of Turin, Grugliasco, Italy

^2^Department of Life Sciences and Systems Biology, University of Turin, Turin, Italy

*** Correspondence:**

Emanuele Pintaldi

[emanuele.pintaldi@unito.it](mailto:emanuele.pintaldi@unito.it)

*Supplementary Table 1. Abundance (%) of plant species in each vegetation type (CC, INT, and SB). Nomenclature followed Pignatti (1982), Flora D’Italia.*

| **Plant species** | **Vegetation type** | | |
| --- | --- | --- | --- |
|  | **CC (%)** | **INT (%)** | **SB (%)** |
| *Agrostis alpina* Scop. | 0.8 | 0 | 0 |
| *Agrostis rupestris* All. | 10.8 | 3.4 | 10.2 |
| *Agrostis schraderiana* Bech. | 0 | 1 | 0 |
| *Alchemilla pentaphyllea* L. | 0.2 | 7.4 | 21.6 |
| *Anthoxanthum alpinum* Löve & Löve | 0.06 | 0 | 0 |
| *Arenaria biflora* L. | 0 | 0 | 0.78 |
| *Bartsia alpina* L. | 1.12 | 1.6 | 0 |
| *Campanula excisa* Murith | 0 | 0.4 | 0.06 |
| *Cardamine bellidifolia* L. subsp. *alpina* (Willd.) Jones | 0.06 | 0 | 0.18 |
| *Carex curvula* All. | 78.4 | 56.4 | 3.06 |
| *Carex parviflora* Host | 0 | 0 | 0.2 |
| *Cerastium cerastoides* (L.) Britton | 0 | 0 | 0.92 |
| *Cerastium uniflorum* Clairv. | 0 | 0 | 0.06 |
| *Cirsium spinosissimum* (L.) Scop. | 0 | 0 | 0.2 |
| *Elyna myosuroides* (Vill.) Fritsch | 0.2 | 0 | 0 |
| *Erigeron alpinus* L. | 0 | 0 | 0.06 |
| *Euphrasia minima* Scleich. | 4.4 | 1.12 | 3.4 |
| *Festuca halleri* All. | 7.6 | 1.2 | 0.06 |
| *Gentiana bavarica* L. | 0 | 0 | 0.12 |
| *Geum montanum* L. | 0 | 0 | 0.2 |
| *Gnaphalium supinum* L. | 1 | 6.4 | 5.06 |
| *Hieracium glanduliferum* Hoppe | 0.4 | 0.2 | 0 |
| *Homogyne alpina* (L.) Cass. | 0 | 0.06 | 0.06 |
| *Leontodon helveticus* Merat | 25 | 20.6 | 0.92 |
| *Leucanthemopsis alpina* (L.) Heyw. | 0.58 | 2.52 | 2.66 |
| *Loiseleuria procumbens* (L.) Desv. | 0.06 | 0 | 0 |
| *Luzula alpino-pilosa* (Chaix) Breistr. | 0 | 1.4 | 0.8 |
| *Minuartia sedoides* (L.) Hiern | 0.26 | 0.2 | 4.06 |
| *Pedicularis kerneri* D. Torre non Huter | 0.26 | 0 | 0 |
| *Phyteuma globulariifolium* Sternb. et Hoppe | 0.4 | 0 | 0 |
| *Phyteuma hemisphaericum* L. | 6.8 | 5.8 | 0.92 |
| *Poa alpina* L. | 1.6 | 4 | 10.6 |
| *Polygonum viviparum* L. | 11 | 0.12 | 0.4 |
| *Ranunculus glacialis* L. | 0.06 | 0 | 0.2 |
| *Sagina saginoides* (L.) Karsten | 0 | 0 | 2.4 |
| *Salix herbacea* L. | 6 | 34.2 | 77.4 |
| *Saxifraga bryoides* L. | 0.06 | 0 | 0 |
| *Saxifraga seguieri* Sprengel | 0 | 0 | 0.06 |
| *Sedum alpestre* Vill. | 0.06 | 0.06 | 0.18 |
| *Senecio halleri* Dandy | 3.4 | 0.18 | 0.06 |
| *Silene acaulis* (L.) Jacq. subsp. *exscapa* (All.) Br.-Bl. | 0.2 | 0.06 | 0.06 |
| *Taraxacum alpinum* s. l. | 0 | 0 | 0.06 |
| *Valeriana celtica* L. | 4 | 0.2 | 0 |
| *Veronica alpina* L. | 0 | 0 | 1.78 |

*Supplementary Table 2. Physical and chemical properties and soil types (IUSS Working Group WRB) in the 5 sites (1, 3, 7, 8, 10).*

| **Site ID** | **Cover Type** | **Horizon** | **Textural Class** | **pH** | **TOC**  **(g kg ^-1^)** | **TN**  **(g kg ^-1^)** | | **C/N** | **Classification WRB** |
| --- | --- | --- | --- | --- | --- | --- | --- | --- | --- |
| **SB1** | **SB** | Ah | n.a. | 5.2 | 27.9 | 2.8 | 10 | | Eutric Skeletic Regosol (Arenic) |
|  |  | A | S | 5.7 | 7.2 | 0.9 | 8 | |  |
|  |  | AC | S | 5.7 | 4.6 | 0.6 | 8 | |  |
|  |  | BC | LS | 5.9 | 6.5 | 0.7 | 9 | |  |
| **SB3** | **SB** | Ah | LS | 5.0 | 13.2 | 1.2 | 11 | | Distric Skeletic Cambisol |
|  |  | BA | LS | 5.0 | 8.4 | 0.8 | 10 | |  |
|  |  | BW | LS | 5.0 | 7.9 | 0.8 | 10 | |  |
| **SB7** | **SB** | Ah | LS | 4.2 | 54.0 | 3.6 | 15 | | Dystric Skeletic Cambisol |
|  |  | AB | LS | 4.8 | 8.0 | 0.7 | 12 | |  |
|  |  | Bw | LS | 5.4 | 7.0 | 0.6 | 11 | |  |
| **SB8** | **SB** | Ah | LS | 5.3 | 42.0 | 3.4 | 12 | | Eutric Endoskeletic Regosols (Arenic, Turbic) |
|  |  | A1 | LS | 5.4 | 9.0 | 0.8 | 11 | |  |
|  |  | A2 | SL | 5.7 | 6.0 | 0.6 | 11 | |  |
|  |  | AC1 | LS | 6.1 | 8.0 | 0.6 | 13 | |  |
|  |  | AC2 | LS | 6.1 | 8.0 | 0.6 | 12 | |  |
|  |  | CA | LS | 6.1 | 8.0 | 0.7 | 11 | |  |
| **SB10** | **SB** | Ah/OH | S | 4.6 | 184.0 | 12.3 | 15 | | Skeletic Umbrisol (Arenic) |
|  |  | A | LS | 4.8 | 14.0 | 1.2 | 12 | |  |
|  |  | BC | LS | 5.1 | 10.0 | 0.7 | 15 | |  |
| **INT1** | **INT** | Ah | LS | 4.6 | 50.2 | 4.1 | 12 | | Skeletic Umbrisol (Humic) |
|  |  | A | SL | 4.3 | 31.0 | 2.5 | 12 | |  |
|  |  | AB | SL | 4.9 | 33.2 | 2.4 | 14 | |  |
|  |  | AC | LS | 4.9 | 15.8 | 1.3 | 12 | |  |
| **INT3** | **INT** | Ah | S | 4.8 | 24.0 | 2.2 | 11 | | Skeletic Leptic Umbrisol (Arenic, Humic) |
|  |  | A | LS | 5.1 | 10.2 | 0.9 | 11 | |  |
|  |  | BA | S | 5.1 | 9.6 | 0.9 | 11 | |  |
|  |  | Bw | LS | 5.1 | 25.7 | 1.8 | 15 | |  |
| **INT7** | **INT** | Ah | S | 4.8 | 58.3 | 4.2 | 14 | | Brunic Leptic Umbrisol (Arenic, Humic) |
|  |  | A | LS | 4.4 | 24.6 | 2.0 | 13 | |  |
|  |  | BA | LS | 4.7 | 19.1 | 1.5 | 12 | |  |
| **INT8** | **INT** | Ah | SL | 4.1 | 49.0 | 3.9 | 13 | | Cambic Leptic Umbrisol (Humic) |
|  |  | AB | SL | 4.4 | 37.3 | 2.7 | 14 | |  |
|  |  | Bw | SL | 4.6 | 25.6 | 1.8 | 14 | |  |
|  |  | BAb | SL | 4.6 | 23.8 | 1.7 | 14 | |  |
|  |  | CR | LS | 5.0 | 4.9 | 0.5 | 10 | |  |
| **INT10** | **INT** | Ah/OH | LS | 4.0 | 140.7 | 10.3 | 14 | | Brunic Leptic Umbrisol (Arenic, Humic, Protospodic) |
|  |  | AE1 | LS | 4.1 | 32.5 | 2.8 | 12 | |  |
|  |  | AE2 | LS | 4.2 | 35.8 | 3.1 | 12 | |  |
|  |  | E/Bh | SL | 4.4 | 46.9 | 2.5 | 18 | |  |
|  |  | Bws | LS | 4.9 | 17.4 | 1.0 | 17 | |  |
| **CC1** | **CC** | Ah1 | LS | 4.2 | 99.7 | 7.8 | 13 | | Haplic Umbrisol (Arenic, Hyperdystrict, Hyperumic) |
|  |  | Ah2 | SL | 4.2 | 77.5 | 5.8 | 13 | |  |
|  |  | AB | LS | 4.3 | 30.8 | 2.7 | 11 | |  |
|  |  | BA | LS | 4.6 | 13.5 | 1.3 | 10 | |  |
| **CC3** | **CC** | Ah | LS | 4.5 | 66.3 | 5.4 | 12 | | Cambisol (Arenic, Humic, Areninovic) |
|  |  | A | LS | 4.3 | 20.2 | 1.9 | 11 | |  |
|  |  | BA | S | 6.3 | 15.8 | 1.5 | 11 | |  |
|  |  | Ab | LS | 4.9 | 22.3 | 1.9 | 11 | |  |
|  |  | BAn1 | LS | 4.8 | 17.4 | 1.5 | 11 | |  |
|  |  | Bab2 | LS | 5.3 | 16.1 | 1.3 | 13 | |  |
|  |  | Bw/CR | SL | 5.4 | 18.0 | 1.5 | 12 | |  |
| **CC7** | **CC** | Ah/OH | LS | 5.2 | 164.5 | 11.8 | 14 | | Skeletic Umbrisol (Arenic, Hyperumic) |
|  |  | Ah1 | LS | 4.2 | 50.4 | 3.8 | 13 | |  |
|  |  | Ah2 | LS | 4.9 | 56.2 | 3.6 | 16 | |  |
|  |  | AC | LS | 4.4 | 45.5 | 3.0 | 15 | |  |
|  |  | A/CR | LS | 4.4 | 30.6 | 2.2 | 14 | |  |
| **CC8** | **CC** | Ah | LS | 4.6 | 100.4 | 7.0 | 14 | | Skeletic Leptic Umbrisol (Arenic, Humic) |
|  |  | AB | LS | 4.6 | 36.2 | 2.9 | 12 | |  |
|  |  | CA | LS | 5.1 | 25.2 | 2.0 | 13 | |  |
| **CC10** | **CC** | Ah | LS | 4.0 | 47.0 | 3.7 | 13 | | Skeletic Leptic Cambisol (Humic) |
|  |  | Bw | LS | 5.0 | 26.2 | 2.1 | 12 | |  |


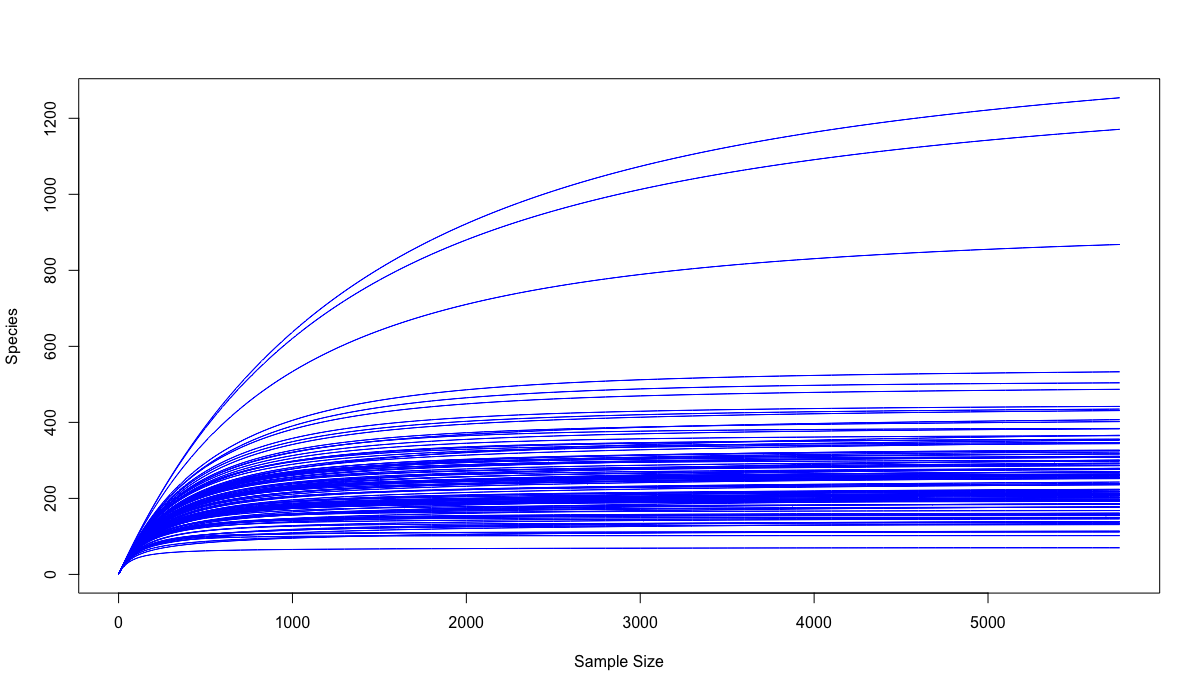


*Supplementary Figure 1. Rarefaction curves of observed ASVs for all samples, illustrating the relationship between sequencing depth and observed species richness.*


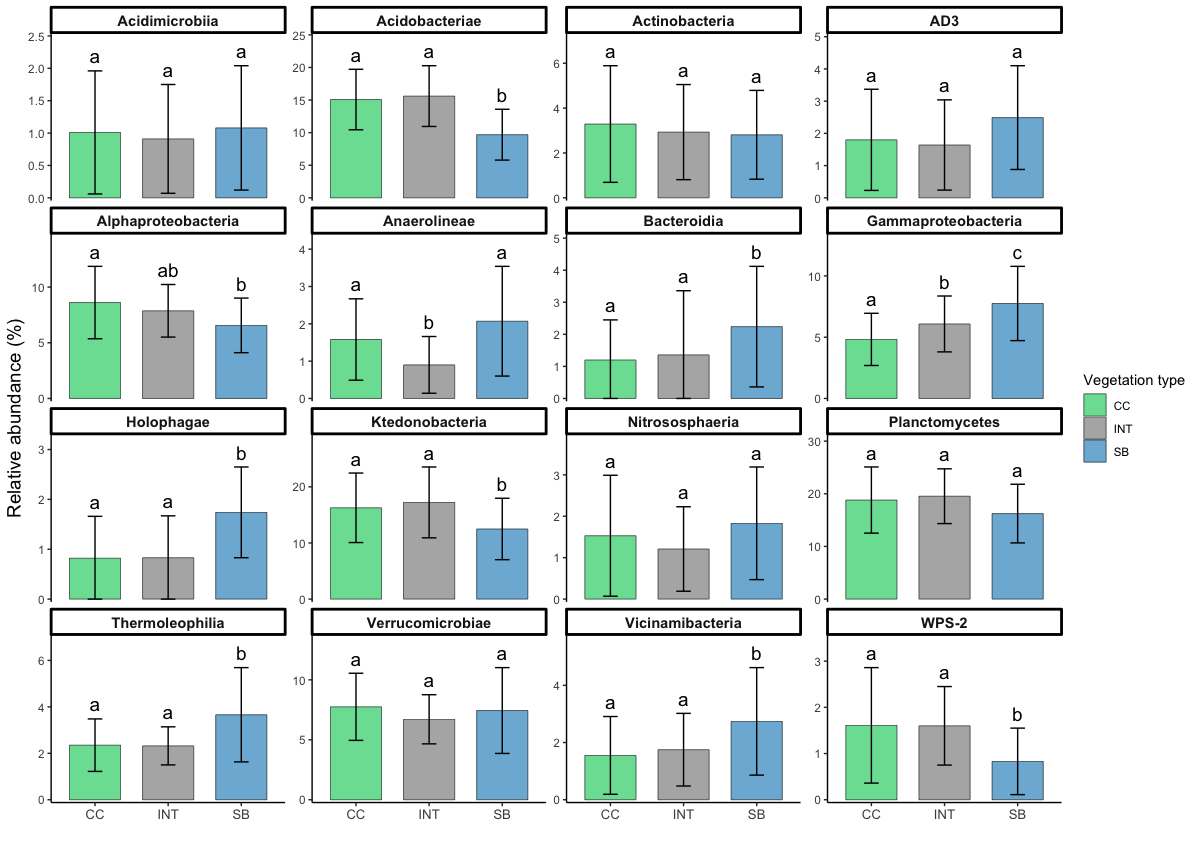


*Supplementary Figure 2. Relative abundance (%) of dominant bacterial classes across vegetation types (CC, green; INT, grey; SB, blue). Bars represent mean ± SD of relative abundance. Only classes with mean relative abundance >1% across samples are shown. Different letters indicate significant differences among vegetation types (Kruskal-Wallis test followed by Dunn’s post hoc test with Benjamini-Hochberg correction, p < 0.05).*
